# Associations Between Active Commuting and Sickness Absence in Finnish Public Sector Cohort of 28 485 Employees

**DOI:** 10.1111/sms.70001

**Published:** 2024-12-14

**Authors:** Essi Kalliolahti, Kia Gluschkoff, Timo Lanki, Jaana I. Halonen, Paula Salo, Tuula Oksanen, Jenni Ervasti

**Affiliations:** ^1^ Finnish Institute of Occupational Health Helsinki Finland; ^2^ Institute of Public Health and Clinical Nutrition University of Eastern Finland Kuopio Finland; ^3^ Department of Public Health Finnish Institute for Health and Welfare Kuopio Finland; ^4^ Department of Environmental and Biological Sciences University of Eastern Finland Kuopio Finland; ^5^ Department of Psychology, Stress Research Institute Stockholm University Stockholm Sweden; ^6^ Department of Psychology and Speech‐Language Pathology University of Turku Turku Finland

**Keywords:** active commuting, cohort study, cycling, occupational health, physical activity, prospective study, sickness absence, walking

## Abstract

Active commuting can be beneficial for health. We examined whether active commuting by walking or cycling was associated with a lower risk of sickness absence in a Finnish public sector cohort of 28 485 employees. We used negative binomial regression to test associations of weekly active commuting in kilometers (no, low, moderate, and high dose) with all‐cause sickness absence. Sickness absence data from employers registers comprised the number of (1) sickness absence days, (2) short (1–9 days) and (3) long (≥ 10 days) sickness absence episodes during 12‐ and 24‐month follow‐ups. The models were adjusted for sociodemographic factors, lifestyle risk factors, and previous sickness absence. To demonstrate absolute risk, we calculated sex‐ and age‐adjusted incidence for sickness absence per 100 person years for each active commuting exposure group. The associations of cycling and walking were additionally studied in separate analyses. Compared to passive commuters (no active commuting), high dose of active commuting (mean of 61 km/week) was associated with an 8%–12% lower relative risk of sickness absence days and an 18% lower relative risk of long episodes. The absolute rate of sickness absence per 100 person‐years was up to 452 days and 10 long episodes lower in the high‐dose active commuters group. In the further analyses separating cyclists and walkers, similar reduced risks were observed only among high‐dose cyclists. Our findings suggest that regular active commuting by bicycle has potential for reducing sickness absence by reducing the risk of long sickness absence episodes.

## 1 Introduction

Insufficient physical activity among the working‐age population is a major public health concern [1, 2]. Active commuting to work provides a practical means to incorporate more physical activity into the workweek [3]. Additionally, emissions from motorized commuting traffic contributes to climate change [4, 5, 6]. To tackle both of these challenges, governments and cities around the world are promoting the use of active and sustainable commute modes such as walking and cycling [4, 6].

The objective health benefits of active commuting, such as lower risk of obesity and diabetes [7], cardiovascular disease incidence [7, 8], and mortality [8, 9] have been established in large prospective cohort studies. Additionally, active commuting has potential for improving and maintaining employee health [10, 11] and work ability [12, 13], and it could also reduce sickness absence. Sickness absence is an important public and occupational health measure, with long‐term sickness absence in particular serving as a strong predictor of permanent labor market exit [14, 15, 16] and mortality [17, 18].

Existing evidence indicates that leisure‐time physical activity associates with lower risk of sickness absence [19, 20, 21, 22]. However, research specifically examining active commuting is limited [23, 24, 25]. Two earlier studies have shown an association between commuting by bicycle and lower sickness absence days [23, 24], but one of them relied on self‐reported sickness absence data [24] and the other was cross‐sectoral [23]. While recent longitudinal evidence from Finland suggests that increased walking or cycling commuting can enhance both employee self‐rated health [11] and work ability [13], some studies indicate that recreational physical activity of vigorous intensity may be required to reduce all‐cause sickness absence [19, 20, 21]. Cycling commuting may be of moderate‐to‐vigorous intensity [23, 26], but walking is typically considered as rather low‐effort activity [26]. However, the associations of active commuting with health benefits may differ from those of voluntary activities due to the habitual nature of commuting, which also leads to an accumulation of commuting experiences [27]. In addition to the objective characteristics of daily commute such as duration and mode, the subjective commute experiences may influence perceived stress and overall wellbeing [27, 28], potentially affecting sickness absence [29].

The aim of this prospective study was to examine whether engaging in active commuting by walking or cycling at varying weekly doses is linked to risk of objectively measured all‐cause sickness absence days and episodes of different lengths after adjusting for several possible confounders.

## 2 Methods

### 2.1 Study Population

**FIGURE 1:**
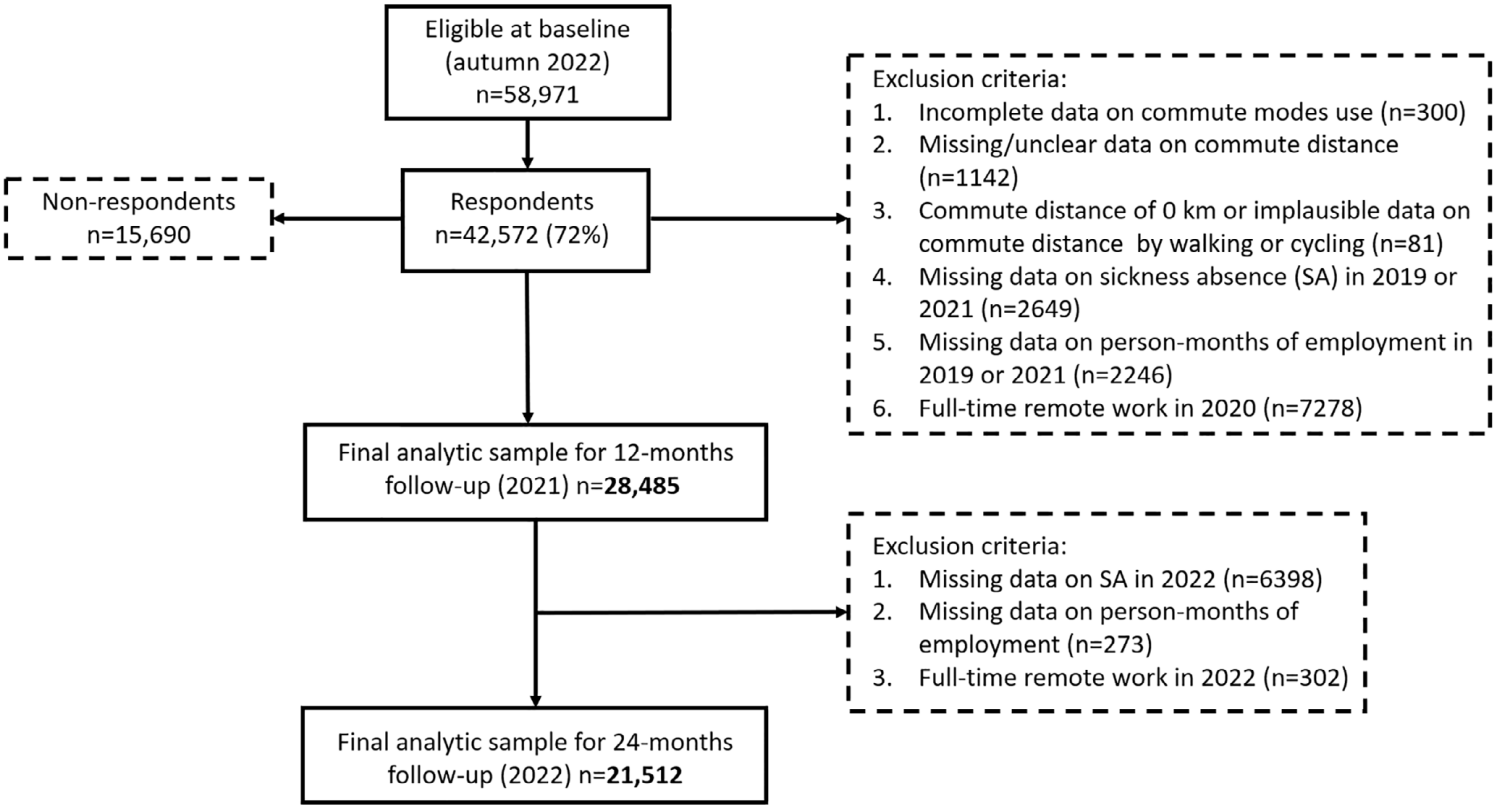
Flow chart of the selection of study populations.

The study population consisted of public sector employees working in the service of four large Finnish cities and who had answered to the Finnish Public Sector (FPS) survey [30] in 2020 including questions on commuting behavior. The response rate was 72% (*n* = 42 572). FPS survey data were linked with employer register data on sickness absence from January 1, 2019 until December 31, 2022. Respondents with missing information on their commute mode use (*n* = 300), missing or unclear information on commute distance (*n* = 1142), and those with no commute (distance of 0 km; *n* = 418) or with an unrealistic regular one‐way commute distance by walking (> 20 km) or by cycling (> 100 km) (*n* = 81) were excluded. Also, those with incomplete data on sickness absence and/or person‐years of employment from 2019 (for adjustments) and/or from 2021 (follow‐up period) (*n* = 4895) and those working from home full‐time/almost full‐time in 2020 (*n* = 7656) were excluded. Sickness absence data from 2022 were available for three participating cities only. With 24‐month follow‐up, we additionally exluded those with missing data on sickness absence and/or person‐years of employment from 2022 (n = 6671) and full‐time or almost full‐time remote workers in 2022 (*n* = 302). The number of participants in the analytic sample with 12‐month follow‐up was 28 485 and with a 24‐month follow‐up it was 21 512. Flow chart of the selection of study population is shown in Figure 1.

### 2.2 Exposure

Our exposure was the average weekly kilometers (km) of active commuting (walking or cycling). Commuting behavior was assessed by asking how many times per week the participant used each commute mode (walking; cycling; public transport; private car use). Questions were asked separately for summer and winter conditions. Participants were also asked to report their one‐way commute distance (km). The reported frequency of walking or cycling was converted to represent the average days per workweek of active commuting: “daily or almost daily” = 5 days; “a few times a week” = 3 days; “once a week” = 1 day; “less than once a week” = 0.5 days; “never” = 0 days. The reported days for summer and winter were added up and then divided by two to represent the average days for the entire calendar year. If the final number of days was 0.5 or less, commuting behavior was considered as passive and replaced with 0 days (*n* = 3467). To produce the measure of average weekly kilometers of active commuting, the days were multiplied by the two‐way commute distance.

Active commuters were categorized into tertiles based on the dose of active commuting: (1) low (≤ 16 km/week), (2) moderate (16 < km/week ≤ 30), and (3) high (> 30 km/week). Same method was applied for assigning participants to groups based on the average weekly kilometers of commuting by walking and cycling separately. The groups for walking were constructed by categorizing the participants with at least some commuting by walking into: (1) low (< 10 km/week), (2) moderate (10 ≤ km/week ≤ 16.5), and (3) high (> 16.5 km/week) dose. Accordingly, the tertiles for cycling commuters were: (1) low‐dose (≤ 16.5 km/week), (2) moderate‐dose (16.5 < km/week ≤ 35), and (3) high (> 35 km/week) dose.

### 2.3 Outcomes

Our outcome was all‐cause sickness absence measured as (1) the number of sickness absence days; (2) the number of short (1–9 days) sickness absence episodes; and (3) the number of long (≥ 10 days) sickness absence episodes during two follow‐up durations: one spanning 12 months and another covering a 24‐month period. In Finland, the Social Insurance Institution provides sickness absence allowance starting after 10 working days, while employers cover the costs for shorter absence periods.

### 2.4 Covariates

We controlled for potential confounders: sex, age, socio‐economic status (SES; high vs. intermediate or low), marital status (married vs. unmarried or cohabiting), type of job contract (permanent vs. fixed‐term), type of employment (full‐time vs. part‐time), body mass index (BMI; kg/m^2^), smoking (current smokers vs. never/ex‐smokers), alcohol use (at‐risk use vs. no to moderate use), and sickness absence days or episodes in 2019 divided by person‐months of employment in 2019 (before the exposure). Person‐months of employment during the follow‐up period were used to determine the time at risk for sickness absence.

SES was categorized into three groups according to 2001 International Standard Classification of Occupations (ISCO) codes: high (managers and senior specialists such as physicians and teachers), intermediate (specialists, office workers and customer service and health and social care workers) and low (manual workers including construction and cleaning services workers and e.g., practical nurses). Alcohol use was assessed through questions on weekly consumption of alcoholic beverages and drinks and then converted to represent weekly grammes of pure alcohol. Based on sex‐specific thresholds, alcohol consumption was further dichotomised into two categories: no use/moderate use and at‐risk use [31].

### 2.5 Statistical Analyses

Due to an over‐dispersed distribution of our outcome variables, we applied negative binomial regression to examine the associations between the dose of weekly active commuting in kilometers (low‐, moderate‐, and high‐dose groups) and sickness absence. Passive commuting (i.e., no dose) was used as a reference category. Negative binomial regression is a generalized linear model that allows for overdispersion, producing rate ratios (RR) with their 95% confidence intervals (CI) [32]. We calculated RRs for sickness absence days and short and long sickness absence episodes by active commuting exposure groups during the follow‐up. Logarithm of the risk‐time for sickness absence (annual person‐months of employment) was included in the models as an offset variable to account for different lengths of at‐risk time.

Model 1 was adjusted for sex and age. Model 2 was additionally adjusted for SES, marital status, type of job contract, type of employment, BMI, smoking status, and weekly alcohol use. To account for prior sickness absence, Model 3 was additionally adjusted for sickness absence days or episodes in 2019 divided by annual person‐months of employment in 2019. In the analyses including the entire study population (*n* = 28 485), the follow‐up time was up to 12 months (year 2021) and with the smaller subpopulation (*n* = 21 512) it was up to 24 months (years 2021 and 2022).

To demonstrate the absolute sickness absence rate by the dose of active commuting, we calculated sex‐ and age‐adjusted incidence with 95% CIs for sickness absence days and short and long sickness absence episodes per 100 person years for each active commuting exposure group. First, we ran an age‐ and sex‐adjusted regression models (with an offset variable for accounting at‐risk time). Then, we produced adjusted predictions for incidence of sickness absence per 100 person years by using mean age of 46 years and median for sex (women) and with 100 years (1200 months) for risk‐time.

The potential mediating (or confounding) effect of baseline job strain [29, 33] was tested in a sensitivity analysis by including it to the models as an additional covariate. The job strain measure was constructed by dividing mean response scores to FPS survey questions on psychological job demands (five items) with mean response scores on skills discretion and decision authority (job control; nine items) so that higher value indicated higher strain [34]. This was further dichotomised into high job strain (high demands and low control; based on study‐specific median split) versus no job strain (all other categories) [33].

To address the potential issues related to very long commutes, including limitations in participating active commuting and a higher risk of sickness absence [35], we conducted sensitivity analyses in a subset of participants with a maximum one‐way commute distance of 10 km (*n* = 18 213). Due to being unable to adjust our main models for leisure‐time physical activity, further sensitivity analyses were run in a subset of participants with moderate‐to‐high level (14–60 MET‐hours per week) of overall physical activity (*n* = 17 642). The measure consisted of a combination of self‐reported leisure‐time and commuting physical activity [36]. All sensitivity analyses were run only during the 12‐month follow‐up period and the models were constructed as our main models.

We further examined separate associations of the dose of commuting by cycling and walking. In the analyses, we used similar four‐category exposure variable of the average weekly kilometers as in our main analyses, and passive commuters (i.e., no dose of cycling nor walking) were used as a reference. From the analyses for cycling, walking commuters with no dose of cycling and from the analyses for walking, cycling commuters with no dose of walking were excluded. The analyses were run for all sickness absence measures with 12‐ and 24‐month follow‐ups. The models were adjusted as our main models.

For all statistical analyses, we used R version 4.2.2. To run the negative binomial regression models, we used an R package MASS [37].

## 3 Results

Descriptive statistics for the entire study population (*n* = 28 485) at baseline (2020) by active commuting exposure groups are shown in Table 1. The mean age of the participants was 46 years, and the majority (79%) of them were women. Descriptives for the subpopulation (*n* = 21 512) that was used in the analyses with up to 24‐month follow‐up are shown in Table S1. The characteristics were largely similar. Table S2 shows more in detail descriptives on active commuting behavior during summer and winter by the dose of active commuting.

**TABLE 1:** The characteristics of the study population (*n* = 28 485) at baseline (2020). Values are counts and percentages unless otherwise stated.

Variable	Total (*n* = 28 485)	Passive (*n* = 17 327)	Low dose (*n* = 3723)	Moderate dose (*n* = 3796)	High dose (*n* = 3639)
Sex					
Women	22 553 (79)	13 509 (78)	3163 (85)	3186 (84)	2695 (74)
Men	5932 (21)	3818 (22)	560 (15)	610 (16)	944 (26)
Age, mean (SD)	46.0 (11.1)	46.3 (11.1)	45.4 (11.3)	45.4 (11.1)	45.7 (10.5)
Marital status					
Unmarried	8967 (32)	5414 (31)	1361 (37)	1245 (33)	947 (26)
Cohabiting	5862 (21)	5862 (21)	722 (19)	794 (21)	790 (22)
Married	13 499 (48)	13 499 (48)	1624 (44)	1737 (46)	1872 (52)
SES					
High	11 562 (45)	6753 (42)	1537 (48)	1664 (49)	1608 (48)
Intermediate	7738 (30)	5053 (32)	841 (26)	863 (26)	981 (29)
Low	6541 (25)	4115 (26)	833 (26)	844 (25)	749 (22)
Job contract					
Permanent	24 274 (85)	14 845 (86)	3098 (83)	3229 (85)	3102 (85)
Fixed term	4211 (15)	2482 (14)	625 (17)	567 (15)	537 (15)
Employment type					
Full‐time	28 019 (99)	17 037 (99)	3672 (99)	3741 (99)	3569 (98)
Part‐time	381 (1)	235 (1)	44 (1)	45 (1)	57 (2)
BMI, mean (SD)	26.5 (5.0)	27.0 (5.2)	26.3 (4.9)	25.6 (4.4)	25.1 (3.9)
Smoking					
No	24 819 (88)	14 763 (86)	3232 (87)	3440 (91)	3384 (93)
Yes	3513 (12)	2473 (14)	469 (13)	335 (9)	236 (7)
Alcohol use					
No use to moderate use	27 193 (69)	16 517 (96)	3547 (96)	3637 (96)	3492 (96)
At‐risk use	1174 (4)	738 (4)	154 (4)	144 (4)	138 (4)
Commute distance (one‐way; km), mean (SD)	12.2 (61.5)	16.9 (78.4)	1.9 (1.5)	4.2 (2.5)	9.4 (5.8)
Active commuting (km/week), mean (SD)	12.2 (24.3)	0 (0.0)	9.9 (3.9)	23.6 (4.4)	60.6 (37.5)

Abbreviation: SD, standard deviation.

Compared to passive commuters, high‐dose active commuters had a lower rate of sickness absence days in the 12 month‐follow‐up (RR = 0.93, 95% CI = 0.87–0.99) and in the 24 month‐follow‐up (RR = 0.89, 95% CI = 0.84–0.95). Similar associations were observed for long sickness absence episodes in both 12‐month (RR = 0.85, 95% CI = 0.77–0.93) and 24‐month (RR = 0.85, 95% CI = 0.78–0.93) follow‐ups. For short episodes, the corresponding estimates were RR = 0.97, 95% CI = 0.92–1.01 and RR = 0.96, 95% CI = 0.92–1.00. In contrast to the results for other sickness absence measures, low‐dose active commuting was associated with a higher rate of short episodes compared to passive commuting in both shorter and longer follow‐up periods (RR = 1.06, 95% CI 1.01–1.11 and RR = 1.04, 95% CI 1.00–1.09, respectively) (Table 2).

**TABLE 2:** Rate ratios (RR) with 95% confidence intervals (CI) for subsequent sickness absence (SA) days and short (1–9 days) and long (≥ 10 days) episodes in active commuting exposure groups during a 12‐ and a 24‐month follow‐up.

Measure of sickness absence (SA)	Active commuting (km/week)	12‐month follow‐up			24‐month follow‐up		
		Model 1	Model 2	Model 3	Model 1	Model 2	Model 3
		RR 95% CI	RR 95% CI	RR 95% CI	RR 95% CI	RR 95% CI	RR 95% CI
SA days	Passive (reference)	1	1	1	1	1	1
	Low dose	0.98 0.92–1.04	0.99 0.92–1.06	1.00 0.94–1.07	0.96 0.91–1.01	1.00 0.95–1.07	1.02 0.96–1.08
	Moderate dose	0.90 0.84–0.96	0.98 0.92–1.05	0.99 0.93–1.06	0.88 0.84–0.93	0.96 0.90–1.01	0.96 0.91–1.01
	High dose	0.81 0.76–0.86	0.89 0.83–0.95	0.93 0.87–0.99	0.78 0.74–0.83	0.87 0.82–0.92	0.89 0.84–0.95
Short SA episodes (1–9 days)	Passive (reference)	1	1	1	1	1	1
	Low dose	1.07 1.22–1.11	1.08 1.04–1.14	1.07 1.03–1.13	1.05 1.01–1.09	1.06 1.01–1.10	1.05 1.01–1.10
	Moderate dose	0.95 0.91–0.99	1.00 0.95–1.05	1.02 0.97–1.07	0.96 0.93–1.00	1.00 0.96–1.05	1.02 0.98–1.06
	High dose	0.88 0.85–0.92	0.95 0.90–0.99	0.97 0.92–1.01	0.89 0.85–0.92	0.94 0.90–0.98	0.96 0.92–1.00
Long SA episodes (10 or mode days)	Passive (reference)	1	1	1	1	1	1
	Low dose	0.97 0.89–1.05	0.99 0.90–1.08	0.99 0.90–1.08	0.93 0.86–1.00	0.99 0.91–1.08	0.99 0.91–1.07
	Moderate dose	0.90 0.83–0.98	0.96 0.88–1.05	0.96 0.88–1.06	0.90 0.84–0.97	0.96 0.88–1.04	0.97 0.89–1.05
	High dose	0.76 0.69–0.83	0.85 0.77–0.93	0.85 0.77–0.93	0.76 0.71–0.83	0.85 0.78–0.92	0.85 0.78–0.93

*Note:* Model 1: adjusted for sex and age. Model 2: adjusted for sex, age, SES, marital status, type of job contract, type of employment, BMI, smoking status, and weekly alcohol use. Model 3: adjusted for sex, age, SES, marital status, type of job contract, type of employment, BMI, smoking status, weekly alcohol use, and sickness absence days or episodes in 2019 divided by annual person‐months of employment in 2019.

**FIGURE 2:**
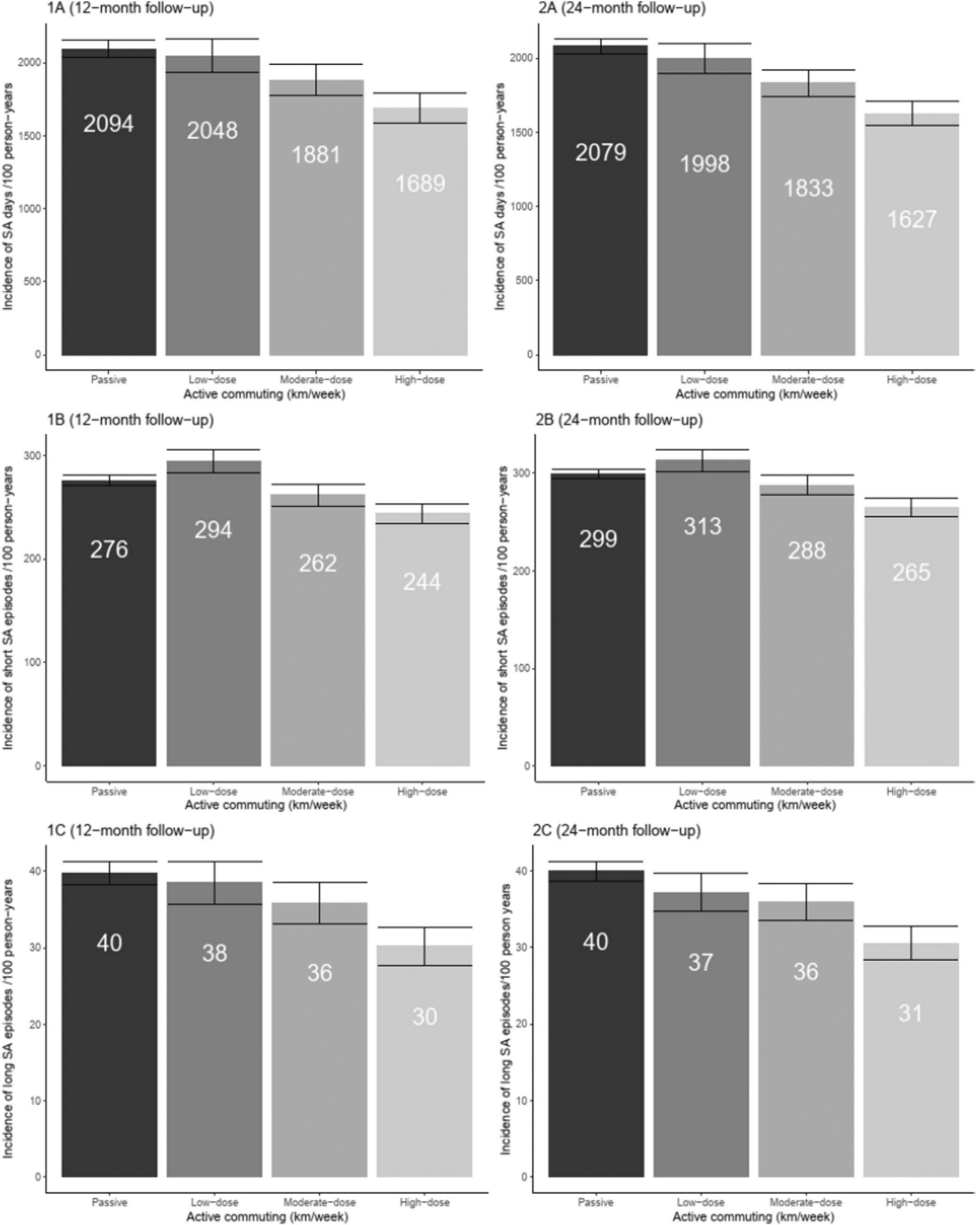
Incidence for sex‐ and age‐adjusted sickness absence (SA) with 95% confidence intervals per 100 person‐years according to active commuting exposure groups during a 12‐ and a 24‐month follow‐up.

Figure 2 shows sex‐ and age‐adjusted incidence of all sickness absence outcomes per 100 person‐years according to active commuting exposure groups. During the 12‐month follow‐up, the absolute rate of sickness absence per 100 person‐years was lowest in the high‐dose active commuting group (1689 days and 30 long episodes) and the highest among passive commuters (2094 days and 40 long episodes). The absolute rate of short sickness absence episodes per 100 person‐years was lowest in the high‐dose group (244 episodes), and highest in the low‐dose group (294 episodes). The results for the 24‐month follow‐up were similar to those of the 12‐month follow‐up: the absolute rate of sickness absence per 100 person‐years measured as days and long episodes was lowest in the high‐dose group (1627 days and 31 episodes) and highest among passive commuters (2079 days and 40 episodes). When measured as short episodes, the rate was lowest in the high‐dose group (265 episodes) and highest in the low‐dose group (313 episodes).

With 12‐month follow‐up, the relative risks of sickness absence days (RR = 0.90, 95% CI 0.83–0.97) and long episodes (RR = 0.81, 95% CI 0.72–0.90) in the high‐dose cycling commuting group were lower compared to passive group. No significant associations with short episodes were observed. Compared to passive commuting, walking was associated with higher risk of short sickness absence episodes in low‐ (RR = 1.10, 95% CI 1.03–1.17) and moderate‐dose (RR = 1.07, 95% CI 1.01–1.14) groups. No significant associations of commuting by walking with sickness absence days or long episodes were observed (Table 3). In the smaller populations with 24‐month follow‐up, low dose of both walking and cycling was associated with higher risk of short sickness absence episodes, but apart from this, the results were similar to those with 12‐month follow‐up (Table S3).

**TABLE 3:** Rate ratios (RR) with 95% confidence intervals (CI) for subsequent sickness absence (SA) days and short (1–9 days) and long (≥ 10 days) episodes in cycling commuting (low dose: *n* = 2772; moderate dose: *n* = 2787; high dose *n* = 2687) and walking commuting (low dose: *n* = 1773; moderate dose: *n*= 1962; high dose *n* = 1822) exposure groups during a 12‐month follow‐up. Entirely passive commuters (i.e., no dose of cycling nor walking; *n* = 17 327) were used as a reference group. From the analyses for cycling, walking commuters with no dose of cycling (*n* = 2912) and from the analyses for walking, cycling commuters with no dose of walking (*n* = 5601) were excluded.

Measure of sickness absence (SA)		Cycling (km/week; *n* = 25 573)			Walking (km/week; 22 884)		
		Model 1	Model 2	Model 3	Model 1	Model 2	Model 3
		RR 95% CI	RR 95% CI	RR 95% CI	RR 95% CI	RR 95% CI	RR 95% CI
SA days	Passive (reference)	1	1	1	1	1	1
	Low dose	0.91 0.85–0.98	0.95 0.88–1.03	1.00 0.92–1.08	0.95 0.87–1.04	0.95 0.88–1.03	1.07 0.97–1.18
	Moderate dose	0.84 0.78–0.90	0.94 0.87–1.02	0.94 0.87–1.02	1.03 0.95–1.12	0.94 0.87–1.01	1.01 0.92–1.11
	High dose	0.75 0.70–0.81	0.85 0.78–0.92	0.90 0.83–0.97	1.04 0.96–1.13	0.85 0.78–0.92	1.03 0.94–1.13
Short SA episodes (1–9 days)	Passive (reference)	1	1	1	1	1	1
	Low dose	1.00 0.95–1.05	1.03 0.98–1.09	1.04 0.99–1.10	1.08 1.02–1.14	1.11 1.04–1.19	1.10 1.03–1.17
	Moderate dose	0.90 0.86–0.94	0.98 0.92–1.03	0.99 0.94–1.05	1.08 1.02–1.14	1.08 1.02–1.15	1.07 1.01–1.14
	High dose	0.86 0.82–0.91	0.94 0.89–0.10	0.96 0.91–1.02	1.01 0.96–1.07	1.00 0.94–1.07	1.02 0.96–1.09
Long SA episodes (10 or mode days)	Passive (reference)	1	1	1	1	1	1
	Low dose	0.96 0.88–1.05	0.99 0.90–1.10	0.99 0.90–1.10	0.95 0.85–1.07	1.07 0.94–1.21	1.06 0.94–1.20
	Moderate dose	0.84 0.76–0.92	0.93 0.84–1.03	0.93 0.84–1.03	1.01 0.91–1.13	1.01 0.90–1.14	1.01 0.89–1.13
	High dose	0.70 0.63–0.78	0.81 0.72–0.90	0.81 0.72–0.90	0.98 0.87–1.09	0.96 0.84–1.09	096 0.85–1.09

*Note:* Model 1: adjusted for sex and age. Model 2: adjusted for sex, age, SES, marital status, type of job contract, type of employment, BMI, smoking status, and weekly alcohol use. Model 3: adjusted for sex, age, SES, marital status, type of job contract, type of employment, BMI, smoking status, weekly alcohol use, and sickness absence days or episodes in 2019 divided by annual person‐months of employment in 2019.

The sensitivity analyses where job strain was included in the models as an additional covariate (Table S4), and in subsamples with a maximum of 10 km commute distance (Table S5) and with moderate‐to‐high level of overall physical activity (Table S6) produced largely similar results as our main analyses.

## 4 Discussion

This study examined whether active commuting was associated with the risk of sickness absence measured in days and short (1–9 days) and long (≥ 10 days) episodes in 12‐ and 24‐month follows. After adjustments, and using passive commuting as a reference, the highest dose of active commuting was associated with 8%–12% lower relative risk of sickness absence days and 18% lower relative risk of long episodes. Accordingly, the absolute rate of sickness absence per 100 person‐years during either follow‐up time was lower by 405–452 days and 9–10 long episodes.

The observed association with lower sickness absence days are in line with two earlier studies [23, 24] where bicycle commuting was associated with lower rate of sickness absence equivalent to 1 day per year. The cross‐sectional study by Hendriksen et al. in 1236 Dutch employees demonstrated that, compared to non‐cyclists, regular cycling was associated with less sickness absence days [23]. They also found some evidence of a dose–response pattern: those who cycled longer distances and more frequently had fewer sickness absence days compared to those cycling shorter distances (up to 5 km) three times a week [23]. Both cycling and walking were included in a longitudinal study by Mytton et al. among 691–801 commuters in the UK [24]. In a 2‐year follow‐up, those who sustained commuting by cycling (any dose) reported less sickness absence days compared to non‐cyclists [24].

To gain sufficient weekly amount of active commuting, our findings suggest that cycling is better than walking. High‐dose active commuters (mean of 61 active km/week) with lower sickness absence risk than passive commuters, had a mean one‐way commute distance of 9 km and they gained their dose mostly by cycling. In comparison, low‐dose active commuters (mean of 10 active km/week) with no beneficial associations with sickness absence and with typically shorter commutes (mean one‐way distance of 1.9 km), gained their dose mostly by walking. Moreover, in our further analysis separating cyclists and walkers, the protective association was observed for high dose of cycling (mean of 67 active km/week), but not walking (mean of 34 active km/week). In addition to the typically lower weekly number of active kilometers among walkers, the intensity of walking may be insufficient. Some earlier studies suggest that to reduce sickness absence, high‐intensity leisure‐time physical activity may be needed [19, 20, 38]. With respect to health benefits of walking in general, higher walking pace (i.e., intensity) may be, in fact, of greater importance than the weekly dose [39]. In line with our findings, the study by Mytton et al. observed no significant associations between sustained walking to work (any dose) and sickness absence [24].

Our overall findings imply that participating in rather regular active commuting by bicycle with often longer distance could reduce sickness absence days by reducing the risk of long (≥ 10 days) episodes. With respect to shorter (1–9) episodes, however, we observed diverging results across the analyses, and no evidence of a protective association. Instead, the lowest weekly doses of active commuting—particularly by walking—were associated with higher risk compared to passive commuting. To be able to further examine this, information on the diagnoses behind sickness absence could be useful, considering that the causes of and the risk factors for shorter sickness absence episodes are likely to differ from those of long episodes [40]. The leading causes of long‐term sickness absence in Finland and other Western countries are mental and musculoskeletal disorders [38]. In a previous study in Finnish middle‐aged municipal employees, leisure‐time physical activity was associated with lower risk of long‐term sickness absence due to any cause, as well as specifically for these causes [38]. In general, compared to short episodes, long‐term sickness absence is shown to be a better indicator for overall health and [17, 18] future disability retirement [14, 15, 16].

Adding job strain to the models had no effect on the estimates, implying it acts neither as a mediator nor a confounder. The results of sensitivity analyses where participants with one‐way distance of more than 10 km where excluded, suggest that our main findings were unlikely driven by the long commute distances of passive commuters.

### 4.1 Strengths and Limitations

The strengths of this study lie in the large cohort with high response rate (72%) and its representativeness of the FPS workforce. The separate survey questions on commuting for summer and winter also enabled us to include Nordic wintertime commuting behavior. We used reliable register‐based data on sickness absence days and episodes of different lengths with a prospective design where the outcomes were measured after the exposure. We examined the associations with sickness absence in 12‐month follow‐up, which enabled us to include participants from all four participating cities, and in 24‐month follow‐up with a smaller subpopulation, allowing to assess potential longer‐term effects. We were also able to adjust our models with several potential confounders including prior sickness absence, which is a strong indicator of health status [17, 18] and future work disability [15, 16], and could also contribute to a lower likelihood of engaging in active commuting.

A major limitation in our study is that, in contrast to the two previous studies [23, 24], we were not able to adjust our models for leisure‐time physical activity. Although weekly hours of overall physical activity were assessed at baseline, the measure does not disentangle between leisure‐time and commuting physical activity. To reduce this limitation, we tested the associations among a subgroup of participants with moderate‐to‐high level of overall physical activity. By doing so, we were able to demonstrate that the observed protective associations among the high‐dose active commuters were unlikely stemming from their higher level of leisure‐time physical activity. In addition, this enabled us to exclude the participants with physical disabilities preventing or restricting their participation in physical activities, including active commuting.

Additional limitations include the potential for self‐report bias, as our measurements of active commuting and overall physical activity rely on self‐reports of commuting behavior, commute distance, and weekly hours of physical activity. To avoid such bias, future research should aim at measuring commuting and leisure‐time physical activity by more objective measures, such as accelerometers and other wearable devices. However, this is often not feasible in large scale epidemiological studies. Despite the high survey response rate (72%), some selection bias may also exist. Moreover, our female‐dominated (80%) public sector cohort may limit the generalizability of our findings to private‐sector or male‐dominated occupations. Finally, our study periods did include the COVID‐19 pandemic which may have affected both commuting behavior and sickness absence.

## 5 Conclusions

This study adds to the existing evidence that regular commuting physical activity by bicycle has potential for reducing sickness absence days by reducing the risk of long‐term episodes. Our findings may provide further reasons for employers to encourage the employees to use active commuting. The employers could invest in incentives such as providing bicycle benefit, bicycle parking, and changing rooms with showers.

## Ethics Statement

The Finnish Public Sector study was approved by the Ethical Committee of the Helsinki and Uusimaa Hospital district (HUS/1210/2016).

## Conflicts of Interest

The authors declare no conflicts of interest.

## Supporting information


Tables S1‐S6.

## Data Availability

Research data are not shared.

## References

[1] R. Guthold , G. A. Stevens , L. M. Riley , and F. C. Bull , “Worldwide Trends in Insufficient Physical Activity From 2001 to 2016: A Pooled Analysis of 358 Population‐Based Surveys With 1.9 Million Participants,” Lancet Global Health 6 (2018): e1077–e1086, 10.1016/S2214-109X(18)30357-7. 30193830

[2] K. Nikitara , S. Odani , N. Demenagas , G. Rachiotis , E. Symvoulakis , and C. Vardavas , “Prevalence and Correlates of Physical Inactivity in Adults Across 28 European Countries,” European Journal of Public Health 31 (2021): 840–845, 10.1093/eurpub/ckab067. 34000007 PMC8504996

[3] M. N. Wanjau , Y. Dalugoda , M. Oberai , et al., “Does Active Transport Displace Other Physical Activity? A Systematic Review of the Evidence,” Journal of Transport and Health 31 (2023): 101631, 10.1016/j.jth.2023.101631.

[4] World Health Organization WHO , Walking and Cycling: Latest Evidence to Support Policy‐Making and Practice (Copenhagen: WHO Regional Office for Europe: World Health Organization, 2022).

[5] C. Brand , T. Götschi , E. Dons , et al., “The Climate Change Mitigation Impacts of Active Travel: Evidence From a Longitudinal Panel Study in Seven European Cities,” Global Environmental Change 67 (2021): 102224, 10.1016/j.gloenvcha.2021.102224.

[6] European Environment Agency EEA , Transport and Environment Report 2021. Decarbonising Road Transport—The Role of Vehicles, Fuels and Transport Demand (Luxembourg: European Environment Agency, 2022).

[7] J. Wu , Q. Li , Y. Feng , et al., “Active Commuting and the Risk of Obesity, Hypertension and Diabetes: A Systematic Review and Meta‐Analysis of Observational Studies,” BMJ Globalization and Health 6 (2021): 6, 10.1136/bmjgh-2021-005838. PMC8237743 34172487

[8] M. Dinu , G. Pagliai , C. Macchi , and F. Sofi , “Active Commuting and Multiple Health Outcomes: A Systematic Review and Meta‐Analysis,” Sports Medicine 49 (2018): 437–452, 10.1007/s40279-018-1023-0. 30446905

[9] F. Dutheil , S. Pelangeon , M. Duclos , et al., “Protective Effect on Mortality of Active Commuting to Work: A Systematic Review and Meta‐Analysis,” Sports Medicine 50 (2020): 2237–2250, 10.1007/s40279-020-01354-0. 33034873

[10] N. Jacob , L. L. Munford , N. N. Rice , and J. Roberts , “Does Commuting Mode Choice Impact Health?,” Health Economics 30 (2021): 207–230, 10.1002/hec.4184. 33145835

[11] E. Haukka , K. Gluschkoff , E. Kalliolahti , et al., “Changes in Active Commuting and Changes in Health: Within‐ and Between‐Individual Analyses Among 16 881 Finnish Public Sector Employees,” Preventive Medicine 177 (2023): 107744, 10.1016/j.ypmed.2023.107744. 37871670

[12] E. Kalliolahti , V. Aalto , P. Salo , T. Lanki , J. Ervasti , and T. Oksanen , “Associations Between Commute Mode Use and Self‐Rated Health and Work Ability Among Finnish Public Sector Employees,” Scandinavian Journal of Public Health 52 (2023): 1–8, 10.1177/14034948231159212. PMC11179311 36942325

[13] E. Kalliolahti , K. Gluschkoff , E. Haukka , et al., “Changes in Active Commuting and Changes in Work Ability and Recovery From Work in 16,778 Finnish Public Sector Employees,” Journal of Transport & Health 38 (2024): 38, 10.1016/j.jth.2024.101872.

[14] L. Salonen , J. Blomgren , M. Laaksonen , and M. Niemela , “Sickness Absence as a Predictor of Disability Retirement in Different Occupational Classes: A Register‐Based Study of a Working‐Age Cohort in Finland in 2007‐2014,” BMJ Open 8 (2018): e020491, 10.1136/bmjopen-2017-020491. PMC5942421 29743328

[15] T. Lund , M. Kivimaki , M. Labriola , et al., “Using Administrative Sickness Absence Data as a Marker of Future Disability Pension: The Prospective DREAM Study of Danish Private Sector Employees,” Occupational and Environmental Medicine 65 (2008): 28–31, 10.1136/oem.2006.031393. 17626139

[16] M. Kivimäki , P. Forma , J. Wikstrom , et al., “Sickness Absence as a Risk Marker of Future Disability Pension: The 10‐Town Study,” Journal of Epidemiology and Community Health 58 (2004): 710–711, 10.1136/jech.2003.015842. 15252077 PMC1732858

[17] J. Vahtera , J. Pentti , and M. Kivimaki , “Sickness Absence as a Predictor of Mortality Among Male and Female Employees,” Journal of Epidemiology and Community Health 58 (2004): 321–326, 10.1136/jech.2003.011817. 15026447 PMC1732735

[18] M. H. Kivimäki , J. E. Ferrie , M. J. Shipley , J. Vahtera , and M. G. Marmot , “Sickness Absence as a Global Measure of Health: Evidence From Mortality in the Whitehall II Prospective Cohort Study,” BMJ 327, no. 7411 (2003): 364, 10.1136/bmj.327.7411.364. 12919985 PMC175810

[19] J. Lahti , M. Laaksonen , E. Lahelma , and O. Rahkonen , “The Impact of Physical Activity on Sickness Absence,” Scandinavian Journal of Medicine & Science in Sports 20 (2010): 191–199, 10.1111/j.1600-0838.2009.00886.x. 19486485

[20] J. Lahti , E. Lahelma , and O. Rahkonen , “Changes in Leisure‐Time Physical Activity and Subsequent Sickness Absence: A Prospective Cohort Study Among Middle‐Aged Employees,” Preventive Medicine 55 (2012): 618–622, 10.1016/j.ypmed.2012.10.006. 23064133

[21] R. Lopez‐Bueno , E. Sundstrup , J. Vinstrup , et al., “High Leisure‐Time Physical Activity Reduces the Risk of Long‐Term Sickness Absence,” Scandinavian Journal of Medicine & Science in Sports 30 (2020): 939–946, 10.1111/sms.13629. 31986220

[22] N. Gupta , S. Dencker‐Larsen , C. Lund Rasmussen , et al., “The Physical Activity Paradox Revisited: A Prospective Study on Compositional Accelerometer Data and Long‐Term Sickness Absence,” International Journal of Behavioral Nutrition and Physical Activity 17 (2020): 93, 10.1186/s12966-020-00988-7. 32690043 PMC7370435

[23] I. J. Hendriksen , M. Simons , F. G. Garre , and V. H. Hildebrandt , “The Association Between Commuter Cycling and Sickness Absence,” Preventive Medicine 51 (2010): 132–135, 10.1016/j.ypmed.2010.05.007. 20580736

[24] O. T. Mytton , J. Panter , and D. Ogilvie , “Longitudinal Associations of Active Commuting With Wellbeing and Sickness Absence,” Preventive Medicine 84 (2016): 19–26, 10.1016/j.ypmed.2015.12.010. 26740344 PMC4766368

[25] L. M. Neumeier , M. Loidl , B. Reich , et al., “Effects of Active Commuting on Health‐Related Quality of Life and Sickness‐Related Absence,” Scandinavian Journal of Medicine & Science in Sports 30 (2020): 31–40, 10.1111/sms.13667. 32246792

[26] B. E. Ainsworth , W. L. Haskell , S. D. Herrmann , et al., “2011 Compendium of Physical Activities: A Second Update of Codes and MET Values,” Medicine and Science in Sports and Exercise 43 (2011): 1575–1581, 10.1249/MSS.0b013e31821ece12. 21681120

[27] J. Liu , D. Ettema , and M. Helbich , “Systematic Review of the Association Between Commuting, Subjective Wellbeing and Mental Health,” Travel Behaviour and Society 28 (2022): 59–74, 10.1016/j.tbs.2022.02.006.

[28] L. D. Murphy , H. R. Cobb , C. W. Rudolph , and H. Zacher , “Commuting Demands and Appraisals: A Systematic Review and Meta‐Analysis of Strain and Wellbeing Outcomes,” Organizational Psychology Review 13 (2022): 11–43, 10.1177/20413866221131404.

[29] S. F. Duijts , I. Kant , G. M. Swaen , et al., “A Meta‐Analysis of Observational Studies Identifies Predictors of Sickness Absence,” Journal of Clinical Epidemiology 60, no. 11 (2007): 1105–1115, 10.1016/j.jclinepi.2007.04.008. 17938051

[30] J. Ervasti , V. Aalto , J. Pentti , T. Oksanen , M. Kivimäki , and J. Vahtera , “Association of Changes in Work due to COVID‐19 Pandemic With Psychosocial Work Environment and Employee Health: A Cohort Study of 24 299 Finnish Public Sector Employees,” Journal of Occupational and Environmental Medicine 79 (2021): 233–241, 10.1136/oemed-2021-107745. 34521683

[31] J. Ervasti , M. Kivimäki , J. Head , et al., “Sickness Absence Diagnoses Among Abstainers, Low‐Risk Drinkers and At‐Risk Drinkers: Consideration of the U‐Shaped Association Between Alcohol Use and Sickness Absence in Four Cohort Studies,” Addiction 113 (2018): 1633–1642, 10.1111/add.14249. 29873143 PMC6099368

[32] P. Schober and T. R. Vetter , “Data in Medical Research: Poisson Regression and Negative Binomial Regression,” Anesthesia & Analgesia 132 (2121): 1378–1379, 10.1213/ANE.0000000000005398. 33857979

[33] K. Heikkila , E. I. Fransson , S. T. Nyberg , et al., “Job Strain and Health‐Related Lifestyle: Findings From an Individual‐Participant Meta‐Analysis of 118 000 Working Adults,” American Journal of Public Health 103 (2013): 103–2097, 10.2105/AJPH.2012.301090. PMC4984954 23678931

[34] S. M. Ruokangas , E. Weiste , J. Ervasti , et al., “Job Demands and Job Control Among Occupational Therapists in Public Sector in Finland,” Scandinavian Journal of Occupational Therapy 29 (2022): 69–78, 10.1080/11038128.2020.1849396. 33242265

[35] L. Ma and R. Ye , “Does Daily Commuting Behavior Matter to Employee Productivity?,” Journal of Transport Geography 76 (2019): 130–141, 10.1016/j.jtrangeo.2019.03.008.

[36] T. Leskinen , S. Stenholm , A. Pulakka , J. Pentti , M. Kivimäki , and J. Vahtera , “Comparison Between Recent and Long‐Term Physical Activity Levels as Predictors of Cardiometabolic Risk: A Cohort Study,” BMJ Open 10 (2020): e033797, 10.1136/bmjopen-2019-033797. PMC7045178 32066606

[37] W. Venables and B. Ripley , Modern Applied Statistics With S, 4th ed. (New York: Springer, 2002).

[38] A. Holstila , O. Rahkonen , E. Lahelma , and J. Lahti , “Changes in Leisure‐Time Physical Activity and Subsequent Sickness Absence due to any Cause, Musculoskeletal, and Mental Causes,” Journal of Physical Activity & Health 13 (2016): 867–873, 10.1123/jpah.2015-0442. 27144730

[39] S. Sabia , A. Dugravot , M. Kivimaki , E. Brunner , M. J. Shipley , and A. Singh‐Manoux , “Effect of Intensity and Type of Physical Activity on Mortality: Results From the Whitehall II Cohort Study,” American Journal of Public Health 102 (2012): 698–704, 10.2105/AJPH.2011.300257. 21940933 PMC3489372

[40] G. Hensing , “The Measurements of Sickness Absence–A Theoretical Perspective,” Norsk Epidemiologi 19 (2009): 147–151.

