# Supplementary material for: Associations Between Active Commuting and Sickness Absence in Finnish Public Sector Cohort of 28 485 Employees
Source: Scand J Med Sci Sports. 2024 Dec 14;34(12):e70001. doi: 10.1111/sms.70001 (PMC11645541; doi:10.1111/sms.70001)
Supplement: Supplementary file 1 — Tables S1‐S6. [file SMS-34-e70001-s001.docx]

**Table S1.** The characteristics of the smaller study population (n=21,512) at baseline (2020) with up to 24-months follow up. Values are counts and percentages unless otherwise stated.

| Variable | Total (n=21512) | Passive (n=12964) | Low dose (n=2827) | Moderate dose (n=2939) | High dose (n=2782) |
| --- | --- | --- | --- | --- | --- |
| Sex |  |  |  |  |  |
| Women | 16791 (79) | 10080 (78) | 2398 (85) | 2451 (83) | 2042 (73) |
| Men | 4541 (21) | 2884 (22) | 429 (15) | 488 (17) | 740 (27) |
| Age, Mean (SD) | 46.1 (10.1) | 46.4 (10.8) | 45.4 (11.0) | 45.5 (10.9) | 45.8 (10.3) |
| Marital Status |  |  |  |  |  |
| Unmarried | 6935 (32) | 4142 (32) | 1067 (38) | 981 (34) | 745 (27) |
| Cohabiting | 4387 (21) | 2628 (20) | 531 (19) | 623 (21) | 605 (22) |
| Married | 10070 (47) | 6129 (48) | 1215 (43) | 1317 (45) | 1409 (51) |
| SES |  |  |  |  |  |
| High | 8672 (45) | 4974 (42) | 1180 (49) | 1295 (51) | 1223 (49) |
| Intermediate | 5714 (30) | 3704 (31) | 540 (27) | 653 (26) | 717 (28) |
| Low | 4858 (25) | 3103 (26) | 565 (24) | 611 (24) | 579 (23) |
| Job Contract |  |  |  |  |  |
| Permanent | 18457 (86) | 11211 (86) | 2346 (83) | 2507 (85) | 2393 (86) |
| Fixed term | 3055 (14) | 1753 (14) | 481 (17) | 432 (15) | 389 (14) |
| Employment Type |  |  |  |  |  |
| Full-time | 21204 (99) | 12777 (99) | 2798 (99) | 2895 (99) | 2734 (99) |
| Part-time | 239 (1) | 143 (1) | 25 (1) | 34 (1) | 37 (1) |
| BMI, Mean (SD) | 26.5 (5) | 27.1 (5) | 26.3 (4.9) | 25.6 (4.4) | 25.1 (3.9) |
| Smoking |  |  |  |  |  |
| No | 18759 (88) | 11050 (86) | 2460 (88) | 2664 (91) | 2585 (93) |
| Yes | 2639 (12) | 1849 (14) | 350 (12) | 257 (9) | 181 (7) |
| Alcohol Use |  |  |  |  |  |
| No use to moderate use | 20536 (96) | 12349 (96) | 2691 (96) | 2824 (96) | 2672 (96) |
| At-risk use | 894 (4) | 567 (4) | 118 (4) | 105 (4) | 104 (4) |
| Commute distance (one-way; km), Mean (SD) | 11.9 (69.6) | 16.4 (89.2) | 1.9 (3.9) | 4.2 (2.5) | 9.3 (5.9) |
| Active Commuting (km/week), Mean (SD) | 12.4 (24.6) | 0 (0.0) | 10.0 (3.9) | 23.6 (4.4) | 60.6 (38.2) |

Abbreviations: SD, standard deviation.

**Table S2.** Average weekly days of active commuting throughout the year and active commute mode shares during summer and winter conditions by active commuting exposure groups. Values are counts and percentages unless otherwise stated.

|  | Total (n=28,485) | Passive (n=17,327) | Low dose (n=3723) | Moderate dose (n=3796) | High dose (n=3639) |
| --- | --- | --- | --- | --- | --- |
| Walking or cycling (days/week; throughout the year), Mean (SD) | 1.5 (1.9) | 0.1 (0.2) | 3.6 (1.6) | 3.5 (1.4) | 3.6 (1.3) |
| Walking (days/week; throughout the year), Mean (SD) | 0.7 (1.5) | 0.0 (0.1) | 2.8 (2.0) | 1.8 (1.9) | 0.7 (1.4) |
| Cycling (days/week; throughout the year), Mean (SD) | 0.9 (1.5) | 0.1 (0.1) | 1.3 (1.5) | 2.2 (1.5) | 3.1 (1.5) |
| Walking in summer at least once per week^†^ | 4354 (15) | 67 (0) | 2390 (64) | 1391 (37) | 506 (14) |
| Walking in summer at least a few times a week^‡^ | 3762 (13) | 0 (0) | 2080 (56) | 1240 (33) | 442 (12) |
| Walking in summer daily or almost daily | 2713 (10) | 0 (0) | 1580 (42) | 859 (23) | 274 (8) |
| Walking in winter at least once per week^†^ | 5219 (18) | 5 (0) | 2560 (69) | 1908 (50) | 746 (21) |
| Walking in winter at least a few times a week^‡^ | 4627 (16) | 0 (0) | 2291 (62) | 1708 (45) | 628 (17) |
| Walking in winter daily or almost daily | 3391 (12) | 0 (0) | 1830 (49) | 1203 (32) | 358 (10) |
| Cycling in summer at least once per week^†^ | 8911 (31) | 524 (3) | 2000 (54) | 2999 (79) | 3388 (93) |
| Cycling in summer at least a few times a week^‡^ | 7984 (28) | 0 (0) | 1760 (47) | 2875 (76) | 3349 (92) |
| Cycling in summer daily or almost daily | 5624 (20) | 0 (0) | 984 (26) | 1979 (52) | 2661 (73) |
| Cycling in winter at least once per week^†^ | 3299 (12) | 1 (0) | 557 (15) | 940 (25) | 1801 (50) |
| Cycling in winter at least a few times a week^‡^ | 2847 (10) | 0 (0) | 437 (12) | 775 (20) | 1635 (45) |
| Cycling in winter daily or almost daily | 1739 (9) | 0 (0) | 217 (6) | 503 (13) | 1019 (28) |

Abbreviations: SD, standard deviation. ^†^Cumulative share additionally including participants with reported frequencies of a few times a week and daily or almost daily. ^‡^Cumulative share additionally including participants with reported frequency of daily or almost daily.

**Table S3.** Rate ratios (RR) with 95% confidence intervals (CI) for subsequent sickness absence (SA) days and short (1–9 days) and long (≥10 days) episodes in cycling commuting (low dose: n=2121; moderate dose: n=2156; high dose n=2034) and walking commuting (low dose: n=1343; moderate dose: n=1478; high dose n=1424) exposure groups during a 24-month follow-up. Entirely passive commuters (i.e., no dose of cycling nor walking; n=12,964) were used as a reference group. From the analyses for cycling, walking commuters with no dose of cycling (n=2237) and from the analyses for walking, cycling commuters with no dose of walking (n=4294) were excluded.

|  |  | Cycling (km/week; n=19,275) | | | Walking (km/week; n=17,218) | | |
| --- | --- | --- | --- | --- | --- | --- | --- |
| Measure of sickness absence (SA) |  | **Model 1** | **Model 2** | **Model 3** | **Model 1** | **Model 2** | **Model 3** |
|  |  | **RR 95% CI** | **RR 95% CI** | **RR 95% CI** | **RR 95% CI** | **RR 95% CI** | **RR 95% CI** |
| SA days | **Passive (reference)** | 1 | 1 | 1 | 1 | 1 | 1 |
|  | **Low dose** | 0.91 0.85-0.96 | 0.97 0.91-1.04 | 0.99 0.93-1.06 | 0.93 0.86-1.00 | 1.02 0.94-1.11 | 1.02 0.94-1.11 |
|  | **Moderate dose** | 0.86 0.81-0.91 | 0.95 0.89-1.02 | 0.96 0.90-1.03 | 0.96 0.89-1.03 | 0.96 0.88-1.04 | 0.97 0.89-1.04 |
|  | **High dose** | 0.74 0.70-0.79 | 0.84 0.78-0.90 | 0.88 0.83-0.94 | 0.94 0.87-1.01 | 0.98 0.90-1.06 | 0.95 0.88-1.03 |
| Short SA episodes (1-9 days) | **Passive (reference)** | 1 | 1 | 1 | 1 | 1 | 1 |
|  | **Low dose** | 1.02 0.98-1.07 | 1.05 1.00-1.10 | 1.05 1.00-1.10 | 1.04 0.99-1.10 | 1.08 1.01-1.14 | 1.07 1.01-1.13 |
|  | **Moderate dose** | 0.92 0.88-0.96 | 0.99 0.94-1.04 | 1.00 0.95-1.05 | 1.06 1.01-1.12 | 1.05 0.99-1.11 | 1.05 0.99-1.11 |
|  | **High dose** | 0.86 0.83-0.90 | 0.94 0.89-0.98 | 0.95 0.91-1.00 | 0.99 0.94-1.05 | 0.99 0.93-1.05 | 1.02 0.96-1.08 |
| Long SA episodes (10 or mode days) | **Passive (reference)** | 1 | 1 | 1 | 1 | 1 | 1 |
|  | **Low dose** | 0.90 0.83-0.98 | 0.96 0.88-1.06 | 0.97 0.88-1.06 | 0.93 0.84-1.03 | 1.04 0.93-1.17 | 1.02 0.91-1.15 |
|  | **Moderate dose** | 0.86 0.79-0.94 | 0.94 0.85-1.03 | 0.94 0.86-1.03 | 0.94 0.85-1.04 | 0.98 0.88-1.10 | 0.98 0.88-1.10 |
|  | **High dose** | 0.72 0.66-0.79 | 0.82 0.75-0.91 | 0.83 0.75-0.91 | 0.96 0.87-1.06 | 0.96 0.85-1.07 | 0.96 0.86-1.08 |

Note: **Model 1**: adjusted for sex and age. **Model 2**: adjusted for sex, age, SES, marital status, type of job contract, type of employment, BMI, smoking status, and weekly alcohol use. **Model 3**: adjusted for sex, age, SES, marital status, type of job contract, type of employment, BMI, smoking status, weekly alcohol use, and sickness absence days or episodes in 2019 divided by annual person-months of employment in 2019.

**Table S4.** Rate ratios (RR) with 95% confidence intervals (CI) for sickness absence (SA) days and short (1-9 days) and long (≥10 days) episodes in active commuting exposure groups during the 12-month follow-up after additionally accounting for job strain (high strain; n=5993 vs. no strain; n=23,146).

|  |  | 12-month follow-up |
| --- | --- | --- |
| Measure of sickness absence (SA) | **Active commuting (km/week)** |  |
|  |  | **RR 95% CI** |
| SA days | **Passive (reference)** | 1 |
|  | **Low dose** | 1.00 0.93-1.07 |
|  | **Moderate dose** | 0.99 0.93-1.06 |
|  | **High dose** | **0.91 0.85-0.98** |
|  |  |  |
| Short SA episodes (1-9 days) | **Passive (reference)** | 1 |
|  | **Low dose** | **1.07 1.03-1.13** |
|  | **Moderate dose** | 1.02 0.97-1.07 |
|  | **High dose** | 0.96 0.92-1.01 |
|  |  |  |
| Long SA episodes (10 or mode days) | **Passive (reference)** | 1 |
|  | **Low dose** | 0.99 0.90-1.08 |
|  | **Moderate dose** | 0.96 0.88-1.05 |
|  | **High dose** | **0.84 0.77-0.93** |

Note: Models adjusted sex, age, SES, marital status, type of job contract, type of employment, BMI, smoking status, weekly alcohol use, and sickness absence days or episodes in 2019 divided by annual person-months of employment in 2019.

**Table S5.** Rate ratios (RR) with 95% confidence intervals (CI) for sickness absence (SA) days and short (1-9 days) and long (≥10 days) episodes in active commuting exposure groups during the 12-month follow-up among a subpopulation with a commute distance maximum of 10 km (n=18,213)

|  |  | 12-month follow-up | | |
| --- | --- | --- | --- | --- |
| Measure of sickness absence (SA) | **Active commuting (km/week)** | **Model 1** | **Model 2** | **Model 3** |
|  |  | **RR 95% CI** | **RR 95% CI** | **RR 95% CI** |
| SA days | **Passive (reference)** | 1 | 1 | 1 |
|  | **Low dose** | 0.92 0.86-0.99 | 0.93 0.87-1.01 | 0.96 0.89-1.03 |
|  | **Moderate dose** | 0.85 0.79-0.91 | 0.94 0.86-1.00 | 0.94 0.88-1.02 |
|  | **High dose** | 0.79 0.73-0.85 | 0.87 0.80-0.94 | 0.90 0.83-0.98 |
|  |  |  |  |  |
| Short SA episodes (1-9 days) | **Passive (reference)** | 1 | 1 | 1 |
|  | **Low dose** | 1.02 0.97-1.06 | 1.05 1.00-1.10 | 1.05 1.00-1.10 |
|  | **Moderate dose** | 0.90 0.87-0.95 | 0.97 0.92-1.02 | 0.99 0.94-1.04 |
|  | **High dose** | 0.87 0.82-0.91 | 0.93 0.88-0.99 | 0.96 0.90-1.01 |
|  |  |  |  |  |
| Long SA episodes (10 or mode days) | **Passive (reference)** | 1 | 1 | 1 |
|  | **Low dose** | 0.91 0.84-1.00 | 0.95 0.86-1.04 | 0.95 0.86-1.04 |
|  | **Moderate dose** | 0.84 0.77-0.92 | 0.91 0.82-1.00 | 0.91 0.83-1.01 |
|  | **High dose** | 0.75 0.68-0.84 | 0.84 0.75-0.95 | 0.85 0.75-0.95 |

Note: **Model 1**: adjusted for sex and age. **Model 2**: adjusted for sex, age, SES, marital status, type of job contract, type of employment, BMI, smoking status, and weekly alcohol use. **Model 3**: adjusted for sex, age, SES, marital status, type of job contract, type of employment, BMI, smoking status, weekly alcohol use, and sickness absence days or episodes in 2019 divided by annual person-months of employment in 2019.

**Table S6.** Rate ratios (RR) with 95% confidence intervals (CI) for sickness absence (SA) days and short (1-9 days) and long (≥10 days) episodes in active commuting exposure groups during the 12-month follow-up among a subpopulation with a moderate-to-high level (14–60 MET-hours per week) of overall leisure-time physical activity (n=17,642)

|  |  | 12-month follow-up | | |
| --- | --- | --- | --- | --- |
| Measure of sickness absence (SA) | **Active commuting (km/week)** | **Model 1** | **Model 2** | **Model 3** |
|  |  | **RR 95% CI** | **RR 95% CI** | **RR 95% CI** |
| SA days | **Passive (reference)** | 1 | 1 | 1 |
|  | **Low dose** | 1.01 0.93-1.10 | 1.00 0.92-1.09 | 0.98 0.91-1.07 |
|  | **Moderate dose** | 0.88 0.81-0.95 | 0.94 0.86-1.02 | 0.94 0.87-1.02 |
|  | **High dose** | 0.84 0.77-0.91 | 0.90 0.82-0.99 | 0.92 0.84-1.00 |
|  |  |  |  |  |
| Short SA episodes (1-9 days) | **Passive (reference)** | 1 | 1 | 1 |
|  | **Low dose** | 1.10 1.05-1.16 | 1.11 1.05-1.17 | 1.09 1.03-1.15 |
|  | **Moderate dose** | 0.97 0.92-1.02 | 1.01 0.95-1.06 | 1.03 0.97-1.09 |
|  | **High dose** | 0.94 0.89-1.00 | 0.98 0.92-1.04 | 0.98 0.92-1.05 |
|  |  |  |  |  |
| Long SA episodes (10 or mode days) | **Passive (reference)** | 1 | 1 | 1 |
|  | **Low dose** | 1.05 0.95-1.16 | 1.05 0.94-1.18 | 1.05 0.93-1.17 |
|  | **Moderate dose** | 0.90 0.81-1.00 | 0.92 0.82-1.03 | 0.92 0.82-1.04 |
|  | **High dose** | 0.77 0.68-0.87 | 0.82 0.71-0.93 | 0.82 0.72-0.93 |

Note: Model 1: adjusted for sex and age. Model 2: adjusted for sex, age, SES, marital status, type of job contract, type of employment, BMI, smoking status, and weekly alcohol use. Model 3: adjusted for sex, age, SES, marital status, type of job contract, type of employment, BMI, smoking status, weekly alcohol use, and sickness absence days or episodes in 2019 divided by annual person-months of employment in 2019. The mean level of physical activity (MET-hours per week; a combination of leisure-time and commuting physical activity) among the participants (n=17,642) was 32.70 (passive: 31.60; low dose: 32.2; moderate dose: 34.5; high dose: 37.1)
